# Multifactorial Analysis of Oral Health-Related Quality of Life in Children with Special Health Care Needs: A Case-Control Study

**DOI:** 10.3390/healthcare13080919

**Published:** 2025-04-17

**Authors:** Mohammed Khalil Fahmi, Sakeenabi Basha, Roshan Noor Mohamed, Alaa Redwan, Arwa U. Alsaggaf, Majd Hashim S. Morad, Yasser Eid Al-Thobaiti

**Affiliations:** 1 Department of Restorative Dentistry, Faculty of Dentistry, Taif University, Taif 21944, Saudi Arabia; mkfahmi@tudent.org; 2 Department of Preventive Dentistry, Faculty of Dentistry, Taif University, Taif 21944, Saudi Arabia; roshan.noor@tudent.org; 3 Pediatric Dentistry Division, Preventive Dentistry Department, Faculty of Dental Medicine, Umm Al-Qura University, Makkah 24381, Saudi Arabia; akredwan@uqu.edu.sa; 4 Prosthodontic Division, Oral and Maxillofacial Surgery Department, Faculty of Dental Medicine, Umm Al-Qura University, Makkah 24381, Saudi Arabia; ausaggaf@uqu.edu.sa; 5 Post Graduate, Faculty of Dental Medicine, Umm Al-Qura University, Makkah 24381, Saudi Arabia; majd.morad20@gmail.com; 6 Department of Oral and Maxillofacial Surgery and Diagnostic Sciences, Faculty of Dentistry, Taif University, Taif 21944, Saudi Arabia; ythobaiti@tudent.org

**Keywords:** OHRQoL, oral health impact profile, dental caries, special need children, children without special care needs

## Abstract

**Objectives:** The present study aims to investigate the oral health-related quality of life (OHRQoL) among children with special needs (CSN) and children without special care needs (CWSCN) in Saudi Arabia and to explore the association between various factors, including dental caries status, sociodemographic characteristics, and behavioral factors, with OHRQoL. **Methods:** A cross-sectional descriptive study was conducted. A total of 773 children were examined (257 with CSN and 516 with CWSCN). OHRQoL was assessed using the Modified Oral Health Impact Profile (OHIP). Multivariable logistic regression was used to determine the relationship between the OHIP (mean score) and independent variables. Results: The mean physical impact was 2.5 ± 1.1 and 3.1 ± 1.7 among 6–11 yrs-old and 12–16-yr-old children (*p* = 0.021), respectively. The mean personal satisfaction score was 3.2 ± 1.7 and 2.4 ± 1.1 among CSN and CWSCN (*p* = 0.001), respectively. Children with special needs had a 3.11 (95% CI: 1.23–5.21, *p* = 0.0001) times higher mean OHIP than CWSCN. Male children had a 1.87 (95% CI: 0.12–2.89, *p* = 0.024) times higher mean OHIP than female children. Children whose parents had primary school or less education had a 1.92 (95% CI: 0.17–3.11, *p* = 0.029) times higher mean OHIP than those whose parents had intermediate or higher education. **Conclusions:** The present study showed that children with special needs had a poor OHRQoL with high mean physical impact, pain, and psychological impact scores compared to CWSCN. A strong association was observed between poor OHRQoL and parental education status, poor oral hygiene practices, and use of non-fluoridated toothpaste.

## 1. Introduction

Special need children refer to children with any physical (deafness, blindness), mental, behavioral (attention deficit hyperactivity disorder, autism), congenital (Down’s syndrome), spectrum disorder, developmental (cerebral palsy), cognitive (intellectual disability), emotional, or sensory impairment or limiting condition that requires health care intervention or the use of specialized services or programs [1]. The challenges faced by these individuals are multifaceted, often extending beyond their primary condition to include social, educational, and healthcare disparities. For instance, children with special needs frequently encounter obstacles in accessing quality education, social inclusion, and healthcare services, which can exacerbate their vulnerabilities and limit their possibilities for growth and development [2,3].

One of the most pressing challenges for individuals with special care needs is maintaining optimal health, particularly their oral health. Studies have shown that these children are at a higher risk of developing oral diseases, such as dental caries, periodontal disease, dental trauma, or anomalies in tooth development, due to various factors, including physical, cognitive, and behavioral impairments [4,5,6,7,8]. Additionally, many individuals with special needs rely on medications that can have side effects detrimental to oral health, such as xerostomia (dry mouth), which increases the risk of dental caries [9]. Compromised immunity, often associated with certain conditions, further exacerbates their susceptibility to oral health problems [9]. These challenges are compounded by difficulties in performing routine oral hygiene, accessing dental care, and effectively communicating their needs [10,11].

Oral health is an essential component of overall health and well-being. The impact of poor oral health extends beyond physical discomfort and significantly affects the overall quality of life (QOL) of individuals with special care needs [8,9,10]. Oral health-related quality of life (OHRQoL) is a critical measure that reflects the influence of oral health on an individual’s physical, psychological, and social functions [12]. OHRQoL refers to the impact of oral health conditions on an individual’s physical, psychological, and social functions [9,10,11,12,13,14]. For children with special needs, poor oral health can lead to pain, difficulty in eating, and speech problems, which may hinder their ability to participate in social activities and achieve academic success [1]. Furthermore, the stigma associated with visible dental issues can contribute to low self-esteem and social isolation, further diminishing their QOL [15]. Children with special needs (CSN) are a vulnerable population that often faces unique challenges in maintaining optimal oral health, which can negatively influence their OHRQoL [10,11,14].

Several studies have highlighted the poorer oral health status [10,11] and OHRQoL among CSN compared to children without special care needs (CWSCN) [14,16,17,18,19]. Previous studies conducted in Saudi Arabia have demonstrated significantly higher rates of dental caries and poorer oral hygiene practices among CSN than among CWSCN [7,11,14,18]. However, these findings underscore the importance of addressing the oral health needs of CSN to improve their overall well-being.

While previous research has highlighted poorer oral health outcomes and reduced OHRQoL among CSN [10,11,14,16], the specific factors contributing to these disparities remain inadequately explored. A multifactorial approach is necessary to identify the complex interplay of factors contributing to their OHRQoL.

The present study aims to investigate the OHRQoL among CSN and CWSCN with the null hypothesis of no difference between OHRQoL among CSN and CWSCN and to explore the association between various factors, including dental caries status, sociodemographic characteristics, dietary factors, and oral hygiene practices, with OHRQoL.

## 2. Materials and Methods

Study design, sample size, sample selection, and population included: A cross-sectional descriptive study was conducted among special needs CWSCN in Jeddah City, Saudi Arabia, from January 2024 to December 2024. A total of 773 children were examined (257 with special needs and 516 CWSCN). The sample size was determined based on a pilot study (conducted among 20 children with special needs and 30 CWSCN; the pilot study sample was not included in the final study sample). The sample size of 700 was calculated with a precision of 35% and an error of 5%. The final sample size was rounded to 750 to compensate for non-response bias. A total of 30 schools (6 schools from each region of Jeddah City) were randomly selected for the study.

Sample methods: A two-stage random sampling method was used. First, the schools were selected using the lottery method. From each selected school, the final sampling unit was selected randomly according to the total number of children (both special needs and CWSCN) in each school.

Ethical clearance and informed consent: Institutional Review Board approval was obtained before the start of the study (Ethical clearance number: HAO-02-T-105).

**Figure 1:**
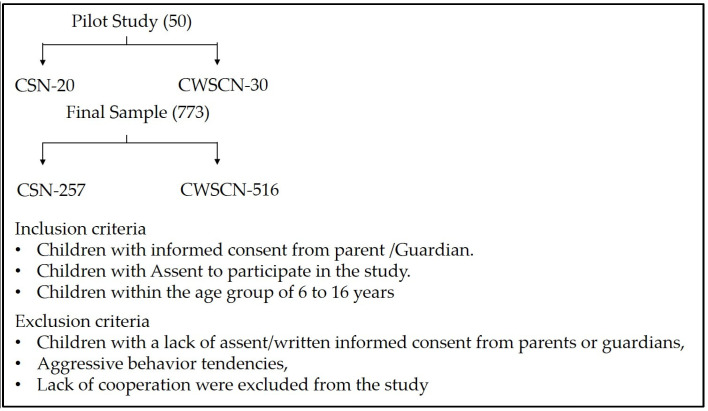
Inclusion and exclusion criteria for subjects included in the study. CSN: Children with special needs; CWSCN: Children without special care needs.

Assent was obtained from the children by explaining what the experience would be, whether it might involve any pain or discomfort, and how long it would take. Children up to 7 years of age were verbally explained what would happen to him/her. For children aged 7 to 12 years, an assent form was used to obtain the child’s willingness to participate in the study. Children aged 13 to 16 years were fully informed about the study, and their assent to participate in the study was obtained. The parents/guardians of the study participants signed written informed consent forms before the child assented. The inclusion and exclusion criteria are summarized in Figure 1.

Sociodemographic, diet, and oral hygiene information: Based on previous studies that showed the impact of sociodemographic, dietary, and oral hygiene practices on dental caries [16,17,18,19], a questionnaire was developed to collect information on the following details:

- Sociodemographic details: age, sex, parents’ education, parents’ occupation, and family income.
- Dietary habits: 72-h recall data, which spanned a weekend and 2 weekdays, number of meals, form, frequency, consistency, and time of sugar intake were recorded.
- Oral hygiene practices: Method of tooth cleaning, material used, frequency of cleaning, and use of fluoridated toothpaste.
- The medication history, previous dental visits, and type and duration of disability were also recorded.

Oral health-related quality of life (OHRQoL): This was assessed using the Modified Oral Health Impact Profile (OHIP) [20]. Parent/caregiver perception about OHRQoL of children’s oral health was collected. It is composed of 14 questionnaires recorded on a five-point Likert scale under three domains (0 = “never”, 1 = “hardly ever”, 2 = “occasionally’, 3 = “fairly often”, and 4 = “very often”):

(1) Physical impact: A total of 9 questions (difficulty in pronunciation, deterioration of taste, diet unsatisfactory due to dental problems, interruption during meals due to dental problems, difficulty in relaxing due to dental problems, difficulty in doing usual jobs due to dental problems, totally unable to function due to dental issues, irritability with others due to dental problems, and less satisfaction in life due to dental issues).
(2) Pain impact: Total of 2 questions (Pain in the mouth; Discomfort while chewing food).
(3) Psychological impact: Total of 3 questions (self-conscious about teeth, mouth, and denture; tensed due to problems of teeth and mouth; embarrassment due to dental issues).

Overall condition of your health: In five categories: 1 = “excellent”, 2 = “very good”, 3 = “good”, 4 = “fair”, 5 = “poor”.

The questionnaire was pretested among 20 special needs children and 30 CWSCN (Cronbach’s α = 0.80).

Categorization of disability: The disability record was taken from school and categorized into 6 groups according to the World Health Organization Criteria [21]: intellectual disability (ID), deafness or blindness or both (DB), autistic disorder (A), Down’s syndrome (DS), cerebral palsy (CP), and multiple disabilities or with syndromes (MD).

Oral examination: All the participants included in the study were examined by a single examiner under natural light using sterile plane mouth mirrors and CPI probes. The World Health Organization (WHO) criteria [22] were used to diagnose dental caries (dmft/dmfs or DMFT/DMFS). Visual and tactile methods were used to examine occlusal lesions, and frank cavitation in interproximal areas was recorded without the use of any radiographs or transillumination. The examiner was trained and calibrated according to the WHO criteria (Kappa value of 0.90, *p* < 0.05, for intra-examiner correlation of dental caries).

Statistical analysis: Differences in means were tested using Student’s *t* test. Multivariable logistic regression was used to determine the relationships between dental caries prevalence (yes/no), age, sex, parental education, dietary factors (frequent sugar consumption, yes/no), oral hygiene factors (tooth brushing frequency and use of fluoridated toothpaste), and OHIP (mean score). The analysis was performed using the Statistical Package for Social Science version 22 (IBM SPSS Statistics, IBM Corp., Armonk, NY, USA). All statistical tests were two-sided, and the significance level was set at *p* < 0.05.

## 3. Result

A total of 773 children were examined (257 special needs and 516 CWSCN). The mean age of the study participants was 10.8 ± 5.2.

Table 1 presents the sociodemographic details according to the health characteristics of the study participants. Of the 257 CSN, 137 (53.3%) were in the 6–11-yrs-age group and 120 (46.7%) were in the 12–16-yrs-age-group. Of the 516 CWSCN, 270 (52.3%) were male and 246 (47.7%) were female.

Table 2 presents the mean OHIP scores among special needs children and CWSCN according to sociodemographic characteristics. The mean physical impact was 2.7 ± 2.0 and 2.1 ± 0.9 among 6–11 years old CSN and CWSCN (*p* = 0.041), respectively. The mean physical impact was 3.5 ± 1.8 and 2.3 ± 0.9 among 12–16-year-old CSN and CWSCN (*p* = 0.031), respectively. The mean pain impact was 3.4 ± 1.9 and 2.9 ± 1.2 among male and female CSN (*p* = 0.043), respectively. Children with parents with primary school education or less had a mean pain impact of 3.1 ± 1.4, and those with parents with intermediate and higher education had a mean pain impact of 2.5 ± 1.1 (*p* = 0.001) in the CSN.

Table 3 presents the mean OHIP among children with special needs and CWSCN according to dietary, oral hygiene, and children’s special needs status. The mean pain impact was 3.1 ± 1.8 and 2.6 ± 1.2 among CSN and CWSCN (*p* = 0.001), respectively. The mean personal satisfaction score was 3.2 ± 1.7 and 2.4 ± 1.1 among CSN and CWSCN (*p* = 0.001), respectively. The mean pain impact was 3.4 ± 1.9 and 1.1 ± 0.1 among caries and caries-free children (*p* = 0.001), respectively. The mean psychological impact was 3.2 ± 1.9 and 2.5 ± 1.6 among children with frequent sugar consumption and those without (*p* = 0.023), respectively. The mean pain impact score was 2.2 ± 1.1 and 2.9 ± 1.6 among children who used fluoridated and non-fluoridated toothpaste (*p* = 0.027), respectively.

The regression analysis results are presented in Table 4. Children with special needs had a mean OHIP score of 3.11 (95% CI: 1.23–5.21, *p* = 0.0001) times higher than that of CWSCN. Male children had a 1.87 (95% CI: 0.12–2.89, *p* = 0.024) times higher mean OHIP than female children. Children whose parents had primary school or less education had a 1.92 (95% CI: 0.17–3.11, *p* = 0.029) times higher mean OHIP than those whose parents had intermediate or higher education. Children with caries had a mean OHIP 2.96 (95% CI: 1.16–5.11, *p* = 0.0001) times higher than that of children without caries. Children who used non-fluoridated toothpaste had a 2.11 (95% CI: 0.91–4.88, *p* = 0.0001) times higher mean OHIP than those who used fluoridated toothpaste.

## 4. Discussion

Oral health disparities and oral health-related quality of life (OHRQoL) among children with special needs (CSN) have been extensively studied across various geographical regions, revealing significant variations in oral health outcomes and their psychosocial impacts [23,24,25,26,27,28,29]. While the challenges faced by CSN are universal, disparities in oral health and OHRQoL among CSN across different regions can be attributed to a range of factors, including socioeconomic status, healthcare infrastructure, cultural attitudes, and the availability of specialized services. In high-income countries, while access to dental care is generally better, socioeconomic inequalities and a lack of tailored services for CSN remain significant barriers. In low-to moderate-income countries, the challenges are more pronounced, with limited resources, inadequate training of healthcare providers, and societal stigma contributing to poorer oral health outcomes and reduced OHRQoL [23,25,27,28,30].

The findings of this study provide a multifaceted understanding of oral health disparities among special needs children (CSN) and CWSCN in Saudi Arabia, emphasizing the interplay between sociodemographic factors, dietary habits, oral hygiene practices, and caries status. The study showed that children with special needs had a poor OHRQoL with a high mean physical impact, pain, and psychological impact scores compared to children without special needs. The results highlight significant disparities in oral health outcomes and their psychosocial impacts, offering critical insights into targeted interventions and policy development.

Questionnaire used to evaluate OHRQoL: Several instruments are used to measure OHRQoL, like Oral Impacts on Daily Performances (OIDP) [31], Geriatric Oral Health Assessment Index (GOHAI) [32], and Oral Health Impact Profile (OHIP) [20,33]. Among these, OHIP is the most widely used by researchers and clinicians [34]. It was originally developed by Slade and Spencer and contained 49 items (OHIP-49) [33]. Shortened versions of this instrument were developed, containing 14 items (OHIP-14) [20]. The study by Campos LA et al. showed high validation of OHIP-14 among dental patients [35]. In the present study, 14 items (OHIP-14) were used.

Disparities Between CSN and CWSCN: CSN consistently exhibited higher oral health impact profile (OHIP) scores across all domains (physical, pain, and psychological) than CWSCN, with higher personal satisfaction scores among CWSCN than among CSN (Table 2 and Table 3). This aligns with studies conducted in Saudi Arabia [11,36], where CSN face barriers such as difficulty performing oral hygiene, limited access to specialized care, and caregiver reliance, exacerbating oral health challenges [11,36]. Similar patterns have been observed globally [10,13,14,16,37], with CSN experiencing higher caries prevalence and poorer quality of life due to systemic neglect of their unique needs [37]. For example, a study in the United States by Lewis et al. (2005) found that children with intellectual disabilities had significantly higher rates of untreated dental caries, leading to pain and functional limitations [38]. Similarly, in low- and middle-income countries, such as India and Yemen, limited access to dental care and a lack of specialized services exacerbate these issues, resulting in poorer oral health outcomes and higher OHIP scores [39,40]. The elevated OHIP scores among CSN underscore the urgency of integrating specialized dental services into Saudi Arabia’s healthcare framework, including caregiver training and mobile clinics, to improve accessibility.

Sociodemographic Determinants: Age, sex, and parental education significantly influenced oral health outcomes. Older children (12–16 years) reported higher physical (*p* < 0.05) and psychological impacts (*p* < 0.05) (Table 2), likely due to increased social awareness, transitioning dentition, and social pressures, indicating that adolescents are more likely to report oral health issues [36]. This mirrors global findings, where adolescents face heightened aesthetic concerns and caries risk [41]. Males had higher physical and pain impact (*p* < 0.05), potentially linked to riskier oral health behaviors, while females reported slightly greater psychological impacts, possibly reflecting societal beauty standards. In contrast, the study conducted by Almajed et.al., [42] showed no significant association between OHRQoL in male and female children. Lower parental education (*p* = 0.029) (Table 4) was associated with poorer oral health outcomes, similar to the findings of past published research [43,44]. Children of parents with lower educational attainment (primary school or less) were more likely to experience negative oral health impacts. This finding is supported by studies emphasizing the role of parental education in shaping children’s oral health behaviors and access to dental care [43,44,45]. Family income plays a pivotal role in shaping oral health outcomes and, consequently, the oral health-related quality of life (OHRQoL) across the globe [46]. The impact of socioeconomic status (SES) on oral health is well documented, with lower-income families experiencing a disproportionate burden of oral disease. This influence is not confined to a single region but manifests in various forms across diverse geographical contexts [47]. However, the present study could not establish a direct influence of family income (*p* > 0.05) (Table 2) on oral health impact, unlike global evidence linking socioeconomic status to reduced access to preventive care and health literacy [48,49]. A review by Almajed O.S et al. highlighted the complex interplay of the impact of socioeconomic factors on pediatric oral health, including oral health-related quality of life [50]. In Saudi Arabia, cultural norms that emphasize familial decision-making may amplify these disparities [51,52], necessitating community-based education programs targeting low-income families.

Impact of Caries and Oral Hygiene Practices: In accordance with previous studies [11,13,14,17], the present study showed that caries status was a critical predictor of OHIP, with affected children reporting significantly higher impacts (Table 3 and Table 4). Frequent sugar consumption (*p* < 0.05) and poor oral hygiene (*p* < 0.05) (≤once daily brushing, non-fluoridated toothpaste) were strongly associated with elevated OHIP scores (Table 3 and Table 4). These findings align with global research emphasizing sugar as a primary caries driver [53] and fluoride’s role in caries prevention [54]. In Saudi Arabia, cultural preferences for sugary diets [55,56] and limited awareness of fluoride benefits [57,58] may exacerbate these issues, highlighting the need for school-based interventions and public health campaigns to promote dietary modifications and the use of fluoridated toothpaste.

Limitations: Due to the cross-sectional nature of the study, it was difficult to assess the causal relationship. The study findings are based on a specific population group that may not fully represent broader demographic diversity. To enhance the generalizability and validity of the results, future research should include a larger and more diverse population to measure the longitudinal impacts of sociodemographic, dietary, oral hygiene, and cultural factors influencing the oral health behaviors of special needs and CWSCN.

## 5. Conclusions

In conclusion, the present study provides valuable insights into the factors influencing the Oral Health Impact Profile (OHIP) among children with special needs (CSN) and CWSCN. The results highlight the significant associations between OHIP and variables such as special needs status, age, sex, parental education, caries status, dietary habits, and oral hygiene practices as key predictors of poor oral health. These findings have important implications for developing targeted interventions to improve oral health outcomes and reduce disparities in children. By addressing sociodemographic barriers, improving preventive care, and fostering collaborative efforts between policymakers and healthcare providers, Saudi Arabia can mitigate these disparities and enhance oral health outcomes for all children.

## Figures and Tables

**Table 1:** Sociodemographic details according to the health characteristics of the study participants.

Variables	CSN (n = 257)	Percentage	CWSCN (n = 516)	Percentage
Age of child				
6–11-yrs	137	53.3	241	46.7
12–16-yrs	120	46.7	275	53.3
Sex				
Male	132	51.4	270	52.3
Female	125	48.6	246	47.7
Parental education				
Primary school or less	45	17.5	77	14.9
Intermediate and high	212	82.5	439	85.1
Parental occupation				
Both parents in job	33	12.8	143	27.7
One of the parent in job	224	87.2	373	72.3
Family income/month				
≤10,000 SR	175	68.1	222	43.0
>10,000 SR	82	31.9	294	57.0

CSN—Children with Special need, CWSCN—Children without special care needs, SR—Saudi riyal.

**Table 2:** Oral health impact profile among children with special needs without special care needs according to sociodemographic characteristics.

Variables	Oral Health Impact Profile (Mean ± SD)			
	Physical Impact	Pain Impact	Psychological Impact	Personal Satisfaction
Age of child				
CSN-6–11-yrs (n = 137)	2.7 ± 2.0	3.1 ± 2.1	2.9 ± 1.8	3.2 ± 1.8
CWSCN-6–11-yrs (n = 241)	2.1 ± 0.9	2.2 ± 0.8	1.3 ± 0.7	2.3 ± 0.9
*t* test, *p* value	0.041	0.032	0.018	0.001
CSN-12–16-yrs (n = 120)	3.5 ± 1.8	3.8 ± 1.9	3.1 ± 2.1	2.9 ± 1.3
CWSCN-12–16-yrs (n = 275)	2.3 ± 0.9	2.4 ± 0.9	2.2 ± 0.9	2.1 ± 0.7
*t* test, *p* value	0.031	0.043	0.052	0.061
Sex				
CSN				
Male (n = 132)	3.3 ± 1.9	3.4 ± 1.9	2.5 ± 1.5	2.7 ± 0.9
Female (n = 125)	2.9 ± 1.3	2.9 ± 1.2	3.0 ± 1.7	2.5 ± 0.8
*t* test, *p* value	0.041	0.043	0.044	0.051
CWSCN				
Male (n = 270)	2.4 ± 1.0	2.7 ± 1.3	2.4 ± 1.1	2.1 ± 0.5
Female (n = 246)	2.2 ± 0.7	2.5 ± 0.9	2.9 ± 1.3	2.4 ± 0.7
*t* test	0.063	0.051	0.042	0.054
Parental education				
CSN				
Primary school or less (n = 45)	3.3 ± 1.6	3.1 ± 1.4	3.4 ± 1.6	2.7 ± 1.1
Intermediate and high (n = 212)	2.4 ± 1.1	2.5 ± 1.1	2.7 ± 1.3	2.2 ± 0.9
*t* test, *p* value	0.001	0.001	0.003	0.053
CWSCN				
Primary school or less (n = 77)	2.8 ± 1.2	2.7 ± 1.3	2.9 ± 1.4	2.6 ± 1.1
Intermediate and high (n = 439)	2.2 ± 0.8	2.1 ± 0.6	2.3 ± 1.1	2.2 ± 0.9
t test, *p* value	0.042	0.046	0.043	0.053
Parental occupation				
CSN				
Both parents in job (n = 33)	3.2 ± 1.6	3.3 ± 1.7	3.1 ±1.7	2.7 ± 1.5
One of the parents in the job (n = 224)	3.1 ± 1.6	2.9 ± 1.3	3.2 ± 1.7	2.3 ± 0.9
*t* test, *p* value	0.062	0.051	0.121	0.073
CWSCN				
Both parents in job (n = 143)	2.4 ± 0.9	2.5 ± 1.1	2.7 ±1.3	2.5 ± 1.1
One of the parents in the job (n = 373)	2.3 ±0.7	2.1 ± 0.7	2.3 ± 1.1	2.1 ± 0.8
*t* test, *p* value	0.062	0.052	0.064	0.063
Family income/month				
CSN				
≤10,000 SR (n = 175)	3.1 ± 1.5	3.2 ± 1.7	2.9 ± 1.5	2.9 ± 1.6
>10,000 SR (n = 82)	2.9 ± 1.2	2.8 ± 1.2	2.6 ± 1.1	2.7 ± 1.1
t test, *p* value	0.052	0.051	0.052	0.063
CWSCN				
≤10,000 SR (n = 222)	2.5 ± 1.3	2.3 ± 1.1	2.4 ± 1.1	2.7 ± 1.1
>10,000 SR (n = 294)	2.3 ± 1.1	2. 1 ± 0.9	2.6 ± 1.2	2.3 ± 0.7
*t* test, *p* value	0.064	0.052	0.071	0.063

CSN—Children with special needs, CWSCN—Children without special care needs, SR—Saudi riyal, SD—standard deviation.

**Table 3:** Oral health impact profile among study participants according to dietary, oral hygiene, caries status, and special needs status.

Variables	Groups	Oral Health Impact Profile (Mean ± SD)			
		Physical Impact	Pain Impact	Psychological Impact	Personal Satisfaction
Special need status	CSN (n = 257)	2.8 ± 1.6	3.1 ± 1.8	2.9 ± 1.4	3.2 ± 1.7
	CWSCN (n = 516)	2.3 ± 1.1	2.6 ± 1.2	2.5 ± 1.1	2.4 ± 1.1
	*t* test	0.021	0.001	0.027	0.001
Caries status	With caries (n = 714)	3.1 ± 1.6	3.4 ± 1.9	3.2 ± 1.8	3.1 ± 1.6
	Caries free (n = 59)	1.2 ± 0.3	1.1 ± 0.1	1.3 ± 1.2	2.1 ± 0.8
	*t* test	0.001	0.001	0.001	0.001
Dietary status	Frequent Sugar consumption (n = 421)	2.7 ± 1.3	2.9 ± 1.4	3.2 ± 1.9	2.9 ± 0.7
	No frequent consumption (n = 352)	2.1 ± 1.1	2.2 ± 1.3	2.5 ± 1.6	2.2 ± 1.1
	*t* test	0.042	0.041	0.023	0.041
Oral hygiene status (Tooth brushing frequency)	≥two times daily (n = 178)	2.2 ± 1.1	2.4 ± 1.1	2.1 ± 0.9	2.2 ± 1.1
	≤once daily (n = 595)	2.7 ± 1.5	2.8 ± 1.5	3.2 ± 1.5	2.7 ± 1.2
	*t* test	0.032	0.041	0.001	0.033
Material used to clean tooth	FT (n = 268)	2.3 ± 1.1	2.2 ± 1.1	2.4 ± 1.1	2.3 ± 0.9
	NFT (n = 455)	2.9 ± 1.5	3.1 ± 1.6	2.7 ± 1.5	2.9 ± 1.2
	Do not know (n = 50)	2.4 ± 1.1	2.3 ± 1.1	2.2 ± 0.9	2.5 ± 1.1
	ANOVA, *p* value	0.031	0.027	0.028	0.031
	Tukey-Post Hoc	NFT > FT	NFT > FT	NFT > FT	NFT > FT

CSN—Children with special needs, CWSCN—Children without special care needs, SD—standard deviation, ANOVA—analysis of variance, NFT—Non-fluoridated toothpaste, FT—Fluoridated toothpaste.

**Table 4:** Multinomial logistic regression analysis with OHIP as the dependent variable.

OHIP	B	Adjusted OR	Lower Bound	Upper Bound	*p* Value
Intercept	−2.312				0.023
CSN	1.172	3.11	1.23	5.21	0.0001
CWSCN #	1.0				
6–11-yr #	1.0				
12–16-yr	0.54	1.02	0.01	1.69	0.053
Male	1.642	1.87	0.12	2.89	0.024
Female #	1.0				
Primary school or less education	1.792	1.92	0.17	3.11	0.029
Intermediate or higher #	1.0				
With caries	1.282	2.96	1.16	5.11	0.0001
Caries free #	1.0				
Frequent Sugar consumption	1.103	1.63	0.08	3.12	0.041
No frequent Sugar consumption #	1.0				
≥two times daily #	1.0				
≤once daily	1.141	1.89	0.12	4.17	0.031
Fluoridated toothpaste #	1.0				
Non-fluoridated toothpaste	0.84	2.11	0.91	4.88	0.0001
Do not know	0.53	0.82	0.01	1.22	0.124

#—Reference value, OR: Odds ratio, OHIP—Oral health impact profile, CSN—Children with special needs, CWSCN—Children without special care needs.

## Data Availability

The original contributions presented in this study are included in this article. Further inquiries should be directed to the corresponding author(s).

## Abbreviations

The following abbreviations are used in this manuscript:

CSN	Children with Special needs
CWSCN	Children without special care needs
OHIP	Oral Health Impact Profile
